# Gefitinib or Erlotinib as Maintenance Therapy in Patients with Advanced Stage Non-Small Cell Lung Cancer: A Systematic Review

**DOI:** 10.1371/journal.pone.0059314

**Published:** 2013-03-21

**Authors:** Xiaofeng Chen, Yiqian Liu, Oluf Dimitri Røe, Yingying Qian, Renhua Guo, Lingjun Zhu, Yongmei Yin, Yongqian Shu

**Affiliations:** 1 Department of Oncology, the First Affiliated Hospital of Nanjing Medical University, Nanjing, China; 2 Department of Cancer Research and Molecular Medicine, Norwegian University of Science and Technology, Trondheim, Norway; 3 Cancer Clinic, Levanger Hospital, Nord-Trøndelag Health Trust, Levanger, Norway; Queen Elizabeth Hospital, Hong Kong

## Abstract

**Background:**

Epidermal growth factor receptor (EGFR) tyrosine kinase inhibitors (TKI), gefitinib and erlotinib have been tested as maintenance therapy in patients with advanced non-small-cell lung cancer (NSCLC). The studies are quite heterogenous regarding study size and populations, and a synopsis of these data could give some more insight in the role of maintenance therapy with TKI.

**Methods:**

In September 2012 we performed a search in the pubmed, EMBASE and Cochrane library databases for randomized phase III trials exploring the role of gefitinib or erlotinib in advanced non-small cell lung cancer. Through a rigorous selection process with specific criteria, five trials (n = 2436 patients) were included for analysis. Standard statistical methods for meta-analysis were applied.

**Results:**

TKIs (gefitinib and erlotinib) significantly increased progression-free survival (PFS) [hazard ratio (HR) 0.63, 95% confidence interval (CI) 0.50–0.76, I^2^ = 78.1%] and overall survival (HR 0.84, 95% CI 0.76–0.93, I^2^ = 0.0%) compared with placebo or observation. The PFS benefit was consistent in all subgroups including stage, sex, ethnicity, performance status, smoking status, histology, EGFR mutation status, and previous response to chemotherapy. Patients with clinical features such as female, never smoker, adenocarcinoma, Asian ethnicity and EGFR mutation positive had more pronounced PFS benefit. Overall survival benefit was observed in patients with clinical features such as female, non-smoker, smoker, adenocarcinoma, and previous stable to induction chemotherapy. Severe adverse events were not frequent. Main limitations of this analysis are that it is not based on individual patient data, and not all studies provided detailed subgroups analysis.

**Conclusions:**

The results show that maintenance therapy with erlotinib or gefitinib produces a significant PFS and OS benefit for unselected patients with advanced NSCLC compared with placebo or observation. Given the less toxicity of TKIs than chemotherapy and simple oral administration, this treatment strategy seems to be of important clinical value.

## Introduction

Current recommendations for chemotherapy treatment of patients with advanced non-small cell lung cancer (NSCLC) are four to six cycles as more cycles do not provide a survival benefit but a higher risk of toxicity [1]. However, only 50–70% patients will have second line treatment, while a substantial proportion of patients do not get further therapy due to side effects or low performance status [2], [3]. Thus, exploration of a non-chemo maintenance strategy has been a sensible development.

Maintenance therapy refers to the use of systemic therapy, either by continuing the primary drug or switch to a new one, in patients who get objective response or stable disease from the first line chemotherapy. This was primarily tested with cytotoxic agents such as gemcitabine [4], docetaxel [3] and pemetrexed [2]. The outstanding results of the JMEN study proved that maintenance of pemetrexed significantly improved the overall survival (OS) in advanced NSCLC patients was a proof of principle [2]. Subsequently, the results of the SATURN study also showed a significant prolongation of progression-free survival (PFS) and overall survival (OS) with maintenance erlotinib compared with placebo [5]. Zhang L et al [6] and other researchers [7], [8] also demonstrated the positive role of maintenance therapy with epidermal growth factor receptor (EGFR) tyrosine kinase inhibitors (TKIs), erlotinib and gefitinib. Due to their low toxicity and good efficacy data, EGFR TKIs have aroused great attention in maintenance therapy. Recently, the updated ASCO guidelines recommended that immediate treatment with an alternative, single-agent chemotherapy (including EGFR TKIs) in patients may be considered [9].

Behera et al [10] carried out a meta-analysis focusing on the role of single agent maintenance therapy in patients with advanced non-small cell lung cancer. They included twelve studies (five meeting abstracts, seven full manuscripts) and showed that single agent maintenance therapy provided superior OS (HR 0.86, 95% CI 0.80–0.92) and PFS (HR 0.62, 95% CI 0.57–0.67). However, only four studies (two meeting abstracts and two full manuscripts) about EGFR TKIs were included. Furthermore, because they emphasized the role of switch and continuation, the outcomes of EGFR TKIs maintenance were not analyzed in detail. Petrelli et al [11] did a pooled analysis of three randomized trials of erlotinib as maintenance therapy and confirmed the addition of maintenance erlotinib significantly improved PFS and OS in patients with advanced non-small cell lung cancer who had not progressed after four cycles of first-line chemotherapy. The benefit seemed to exist across the subgroups. But that analysis did not include any study on gefitinib maintenance.

We thus conducted this meta-analysis of high quality randomized clinical trials on maintenance therapy with gefitinib and erlotinib. Our aim was to determine the role of maintenance EGFR TKIs in patients with advanced NSCLC and to explore which subgroups of patients who will benefit from EGFR TKIs maintenance.

## Patients and Methods

### Search Method

In September 2012, an electronic search of the Pubmed, the EMBASE and the Cochrane library databases was performed. The search keywords were: “gefitinib and maintenance”, or “erlotinib and maintenance”, “non-small cell lung cancer (NSCLC)”. The list of retrieved studies was then manually searched and reviewed. The published languages and years were not limited. Meeting abstracts from the American society of Clinical Oncology (ASCO) (2007–2012) and World Congress of Lung Cancer (WCLC) (2007–2011) were also hand searched for eligible trials. Reference lists of original articles and review articles were also examined for additional literature.

### Selection of Trials

Details on the selection process are shown in the supplementary information file (Protocol S1). The selection of trials were performed by two authors and blinded. Randomized controlled phase III trials reporting the efficacy of gefitinib or erlotinib as maintenance therapy (single or combined with other agents except chemotherapeutics) immediately after the first line chemotherapy in stage IIIB/IV NSCLC were considered eligible. Patients should be pathologically or cytologically diagnosed with NSCLC and randomized just before the maintenance period. Peer-reviewed meeting abstracts fulfilling the criteria were also included. All quality of all eligible studies were also assessed by the Jadad Scale [12]. When a study had updated data, both the original and updated papers or abstracts were included. The references were screened by titles and further selected by reading the abstracts. Papers fulfilling the criteria were reviewed in detail. Articles were also obtained from cross-checking references of publications.

### Data Extraction

Two independent reviewers extracted all data from the identified papers using standardized data compilation forms. Disagreements were resolved by discussion. The methodological quality of each paper was scrutinized and data such as the first author, year of publication, number of patients, median age, percentage of adenocarcinoma and data related to the clinical outcomes such as objective response rate (ORR), progression free survival (PFS), overall survival (OS), adverse events (AE) were extracted. The final data were used when updated data were available. Supplementary data of the IFCT-GFPC 0502 and EORTC 08021/ILCP 01/03 study were obtained by contacting the correspondent authors.

### Statistics

The primary outcome of this systematic review is whether the TKIs maintenance will produce OS benefit, the secondary outcomes including PFS benefit, subgroups analysis regarding OS and PFS, response rate and data on harms. First, the outcome data were pooled and reported as hazard ratio (HR) (fixed model or random model). HR <1 represents results in favor of TKI maintenance therapy. The PFS and OS were estimated by collection of HRs with 95% Confidence Intervals (CIs) which were mentioned in the original publications. The pooled risk ratio (RR) for ORR was calculated from the number of events and the number of patients at risk in each group. Prespecified subgroup analysis according to clinical features was also done.

The study heterogeneity was tested and a P<0.1 was defined as heterogenous. A fixed-effect model (Mantel Haenszel) was applied in case of absence of heterogeneity between studies and otherwise a random-effect model was performed. The meta-analysis results were displayed as forest plots. All calculations were performed using Stata (version 11, Stata, USA).

## Results

### Study Selection Results

**Figure 1:**
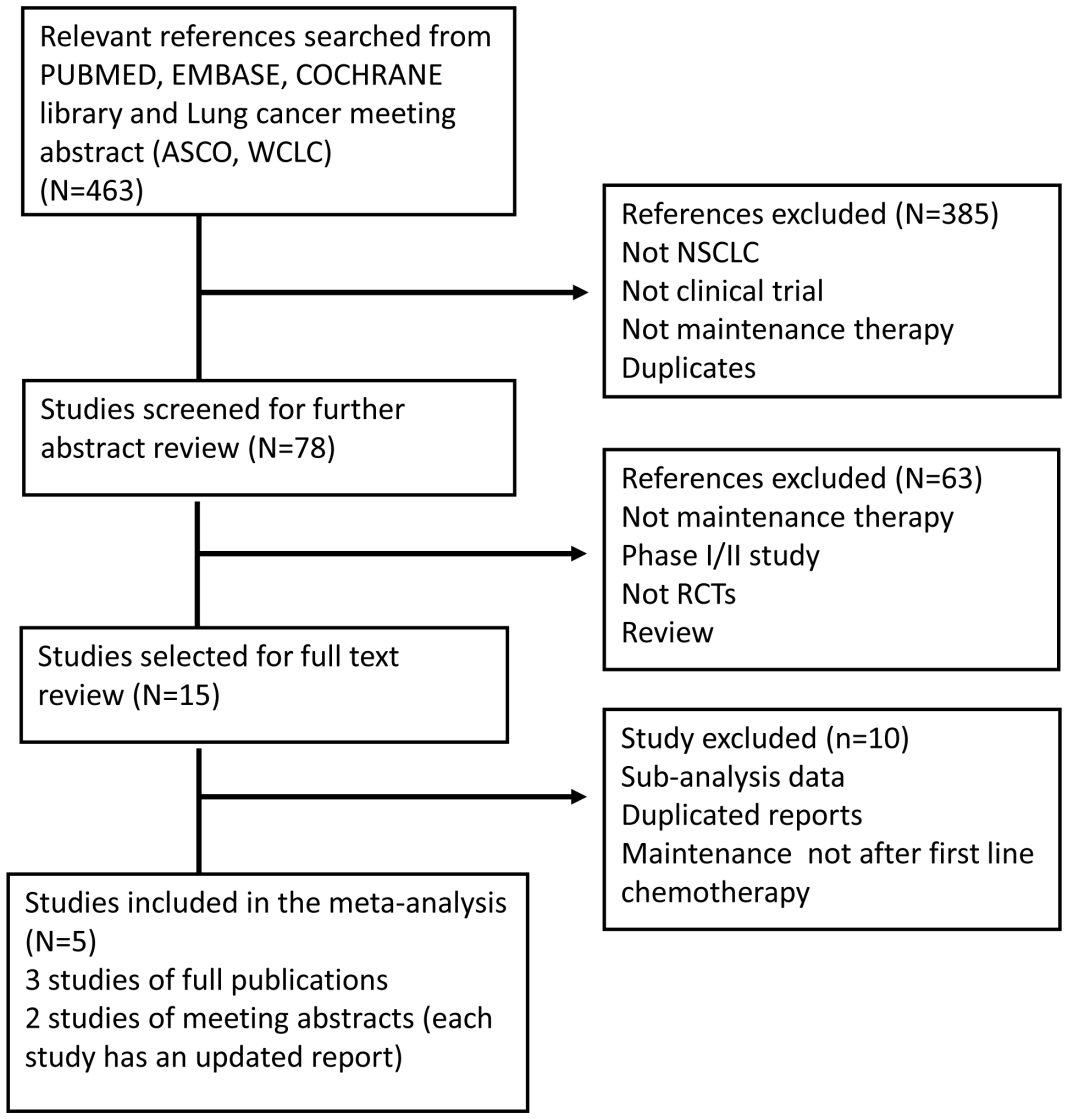
Flow-chart of the meta-analysis.

Our search in electronic database and meeting abstracts retrieved 463 references. Of these, only five studies [5], [6], [7], [8], [13], [14] met the criteria. The selection steps are summarized in the flow chart shown in Fig. 1. One phase III study (WJTOG0203) [15] comparing 3 cycles of platinum-doublet chemotherapy followed by gefitinib maintenance with continued platinum-doublet chemotherapy in Japanese patients with advanced non-small cell lung cancer was excluded because the randomization was performed before the beginning of chemotherapy rather than before the maintenance period and the continuous use of chemotherapy. Another study, SWOG S0023 study [16], evaluated gefitinib maintenance in inoperable stage III NSCLC patients who had not progressed after chemoradiotherapy (concurrent cisplatin and etoposide with thoracic radiation, then 3 cycles docetaxel consolidation). This study was excluded because the maintenance was not after first line chemotherapy and the patients were not stage IIIB/IV.

The data of the final five studies containing a total of 2436 patients were in 7 publications. The results of the SATURN study was formally published in Lancet Oncology in 2010 by Cappuzzo [5], and the data in Asian patients were separately reported in Lung Cancer most recently by Wu [17]. The ATLAS study was initially reported at the 2009 ASCO annual meeting by Miller [14], with updated OS in 2010 by Kabbinavar [7]. The original and updated publications were all included where the PFS and safety data were obtained from the original publications while the OS data were extracted from the updated abstracts.

### Characteristics of the Five Trials in the Review

All of the five trials identified were randomized and controlled phase III trials, and all were of high quality assessed by the Jadad scale (Score4 or more). Two of the studies [6], [8] used gefitinib (250 mg/qd) and the other three used erlotinib (150 mg/qd) maintenance [5], [7], [13]. In all studies maintenance was commenced after 4 cycles’ first line chemotherapy in stage IIIB/IV NSCLC. All of the studies enrolled patients with a mixed population (EGFR mutated and non-mutated) and two of the studies (INFORM and SATURN) reported the outcomes of EGFR patients related to EGFR status. Four studies were double blind and placebo controlled, and only one trial (IFCT-GFPC 0502) [13] was open label. This study investigated maintenance gemcitabine or erlotinib versus observation, following induction chemotherapy. Only the data of erlotinib and observation arms were extracted for the present analysis. A total of 2436 patients were included where the majority were of Caucasian, second largest group were Asian (n = 521) and one study included patients of Arabic decent (n = 54). There were 2391(98%) patients with ECOG performance status 0–1, and 1512(62%) had adenocarcinoma. A summary of the trial characteristics and clinical outcomes is provided in Table 1.

**Table 1:** Summary of characteristics and major results of the included studies.

Studies	First author/year	Numberof Pts	Ethnicity Caucasian/Asian/Other (%)	Median Age	Non-Smoker n (%)	Adenocarcinoma n (%)	Primary endpoint/sign	Exp vs control arms	Known EGFR status n (%)	EGFR mut, Exp/control n (%)	RR (%), Exp vs control, *P*	PFS(m), Exp vs control, *P*	OS(m), Exp vs control, *P*	AE≥Grade3, Exp vs control (%)
INFORM [6]	Zhang L2012	296	0/100/0	55	160 (54%)	209 (71%)	PFS/Yes	G vs placebo	79(27%)	15(10%)/15(10%)	24% vs 1% *P* = 0.0001	4.8 vs 2.6 *P*<0.0001	18.7 vs 16.9 *P* = 0.26	10(7%) vs 5(3%)
EORTC08021/ILCP01/03 [8]	GaafarRM 2011	173	NR	61	38 (22%)	89 (51%)	OS/No	G vs placebo	NR	NR	12% vs 1% *P* = 0.004	4.1 vs 2.9 *P* = 0.0015	10.9 vs 9.4 *P* = 0.2	NR
SATURN [5]	CappuzzoF 2010	889	84/15/1	60	152 (17%)	403 (45.3%)	PFS/Yes	E vs placebo	446(50%)	22(5%)27(6%)	12% vs 5% *P* = 0.0006	12.3 vs 11.3 weeks *P*<0.0001	12 vs 11 *P* = 0.0088	47(11%) vs 34(8%)
IFCT-GFPC0502 [13]	Perol M2012	310	NR	58	29 (9%)	200 (65%)	PFS/Yes	E vs placebo	188(40.5%)†	NR	NR	2.9 vs 1.9 *P* = 0.003	11.4 vs 10.8 *P* = 0.3043	24 (15.5%) vs 4 (2.6%)
ATLAS [7]	Kabbinavar FF 2010	768	78/12/10	64	127 (17%)	609 (82%)	PFS/Yes	E+ Bev vs placebo+ Bev	NR	NR	NR	4.8 vs 3.7 *P* = 0.0012	15.9 vs 13.9 *P* = 0.2686	NR

Abbreviations: Pts, patients; sign, significant; Exp, experimental arm; G, Gefitinib; E, erlotinib; Bev, bevacizumab; PFS, progression free survival in months; OS, overall survival in months; AE, adverse event; NR, not reported.

† This ratio was based on the all included patients in IFCT-GFPC 0502, n = 464.

### Response Rate

Data on response rate was available only in three of the trials, two with gefitinib and one with erlotinib and where the control arms were placebo. The gefitinib studies INFORM and the EORTC showed a higher response rate than the SATURN study [5], [6], [8]. However, the overall response rate was 14.50% (n = 671) in EGFR TKI maintenance and 3.8% (n = 682) in the control arm respectively. The pooled RR was 3.80 (95% CI 2.49–5.79), indicating that EGFR TKIs have a significant tumor shrinking effect after induction with chemotherapy.

### Progression Free Survival

**Figure 2:**
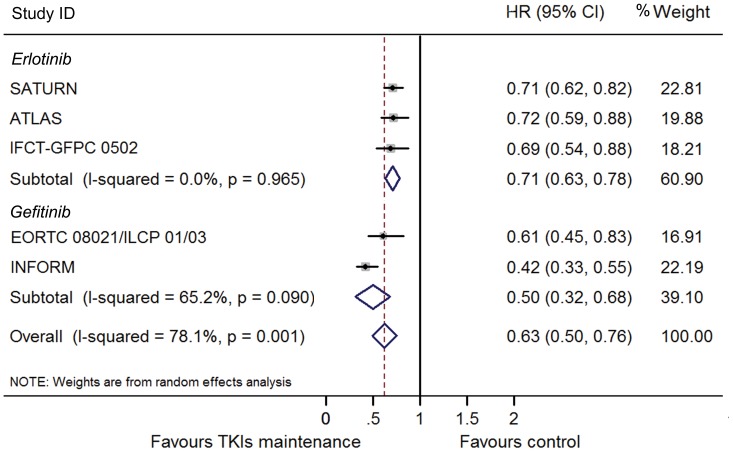
Meta-analysis of hazard ratio (HR) for progression free survival (PFS).

**Figure 3:**
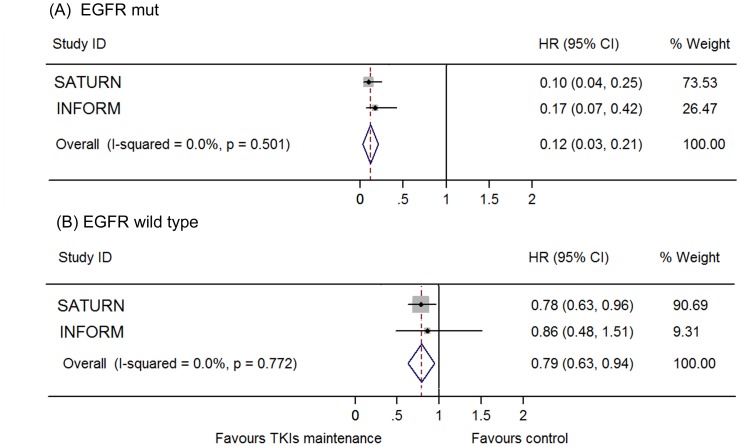
Meta-analysis of hazard ratio (HR) for progression free survival (PFS) according to EGFR mutation status. (A) EGFR mutation positive. (B) EGFR wild type.

TKI maintenance therapy provided significant improvement in PFS (HR 0.63, 95% CI 0.50–0.76; I^2^ = 78.1%; Random model; Fig. 2). The significance is consistent between gefitinib (HR 0.50, 95% CI 0.32–0.68) and erlotinib subgroups (HR 0.71, 95% CI 0.63–0.78). The INFORM study provided a much lower HR (0.42, 95% CI 0.33–0.55) and this may lead to the heterogeneity between the trials. When excluded the INFORM study, the results turned out to be homogenous with a similar favorable HR (0.70, 95% CI 0.63–0.76; I^2^ = 0.0). We also did a cross-study subgroup analysis by pooling the HRs of the common subgroups reported in these studies. The results showed that the maintenance treatment of EGFR-TKIs provided consistent PFS benefits in all subgroups, including stage, sex, ethnicity, PS, smoking status, histology, EGFR mutation status and previous response to chemotherapy (Table 2). Notably, more impressive benefit was found in patients with EGFR mutation (HR 0.12, 95% CI 0.03–0.21) according to SATUN and INFORM study (Fig. 3).

**Table 2:** Summary of meta-analysis of progression free survival (PFS) in subgroups.

Subgroups	Study ID	HR (95% CI)	Weight %	Pooled HR (95% CI), I^2^,model
Stage IIIb	SATURN	0.83(0.62–1.10)	39.57	0.61(0.34–0.88)
	EORTC 08021/ILCP 01/03	0.47(0.20–1.13)	21.37	59.8%, Random
	INFORM	0.46(0.28–0.77)	39.06	
Stage IV	SATURN	0.68(0.58–0.81)	36.9	0.57(0.38–0.76)
	EORTC 08021/ILCP 01/03	0.63(0.45–0.89)	27.09	80.2%, Random
	INFORM	0.41(0.30–0.55)	36.01	
Male	SATURN	0.78(0.66–0.92)	30.43	0.68(0.55–0.82)
	EORTC 08021/ILCP 01/03	0.75(0.58–0.98)	22.07	60.1%, Random
	IFCT-GFPC 0502	0.70(0.52–0.92)	22.07	
	INFORM	0.49(0.35–0.69)	25.43	
Female	SATURN	0.56(0.42–0.76)	28.09	0.52(0.37–0.68)
	EORTC 08021/ILCP 01/03	0.63(0.48–0.83)	27.54	62.0%, Random
	IFCT-GFPC 0502	0.64(0.38–1.08)	13.45	
	INFORM	0.34(0.22–0.51)	30.92	
Asian	SATURN	0.58(0.38–0.87)	27.45	0.40(0.21–0.58)
	ATLAS	0.18(0.06–0.55)	27.45	62.0%, Random
	INFORM	0.42(0.21–0.58)	45.10	
Caucasian	SATURN	0.75(0.64–0.88)	62.52	0.75(0.66–0.85)
	ATLAS	0.76(0.61–0.92)	37.48	0.0%, Fixed
PS = 0	SATURN	0.59(0.45–0.77)	50.67	0.61(0.49–0.72)
	ATLAS	0.65(0.47–0.91)	26.80	0.0%, Fixed
	IFCT-GFPC 0502	0.60(0.39–0.87)	22.52	
PS = 1	SATURN	0.77(0.65–0.92)	52.33	0.75(0.65–0.85)
	ATLAS	0.72(0.57–0.91)	33.00	0.0%, Fixed
	IFCT-GFPC 0502	0.75(0.65–0.85)	14.77	
Non-smoker	SATURN	0.56(0.38–0.81)	18.27	0.40(0.31–0.49)
	EORTC 08021/ILCP 01/03	0.51(0.25–1.02)	5.70	0.0%,Fixed
	ATLAS	0.34(0.19–0.61)	19.15	
	IFCT-GFPC 0502	0.30(0.11–0.81)	6.90	
	INFORM	0.36(0.25–0.51)	49.98	
Smoker	SATURN	0.74(0.58–0.93)	22.38	0.69(0.61–0.78)
	EORTC 08021/ILCP 01/03	0.62(0.43–0.89)	12.96	10.9%, Fixed
	ATLAS	0.76(0.62–0.93)	28.53	
	IFCT-GFPC 0502	0.74(0.58–0.96)	18.99	
	INFORM	0.52(0.35–0.75)	17.14	
Adeno	SATURN	0.60(0.48–0.75)	25.77	0.54(0.38–0.71)
	ATLAS	0.64(0.52–0.80)	25.45	82.0%, Random
	IFCT-GFPC 0502	0.63(0.46–0.86)	21.45	
	INFORM	0.33(0.24–0.46)	27.32	
Non-adeno	SATURN	0.76(0.60–0.95)	58.01	0.77(0.64–0.90)
	ATLAS	0.86(0.44–1.27)	10.31	0.0%, Fixed
	IFCT-GFPC 0502	0.79(0.72–1.08)	16.31	
	INFORM	0.72(0.46–1.14)	15.37	
EGFR mut	SATURN	0.10(0.04–0.25)	73.53	0.12(0.03–0.21)
	INFORM	0.17(0.07–0.42)	26.47	0.0%, Fixed
EGFR	SATURN	0.78(0.63–0.96)	90.69	0.79(0.63–0.94)
wild type	INFORM	0.86(0.48–1.51)	9.31	0.0%, Fixed
Previous	SATURN	0.68(0.56–0.83)	30.75	0.60(0.43–0.76)
SD	EORTC 08021/ILCP 01/03	0.64(0.43–0.97)	18.89	67.8%, Random
	IFCT-GFPC 0502	0.71(0.50–1.02)	19.62	
	INFORM	0.41(0.29–0.56)	30.75	
Previous	SATURN	0.74(0.60–0.92)	39.75	0.65(0.48–0.82)
ORR	EORTC 08021/ILCP 01/03	0.55(0.33–0.91)	12.10	51.5%, Random
	IFCT-GFPC 0502	0.67(0.47–0.94)	18.42	
	INFORM	0.44(0.29–0.66)	29.73	

Abbreviations: HR, hazard ratio; 95% CI, 95% confidence interval; Adeno, adenocarcinoma; SD, stable disease; ORR, objective response rate.

### Overall Survival

**Figure 4:**
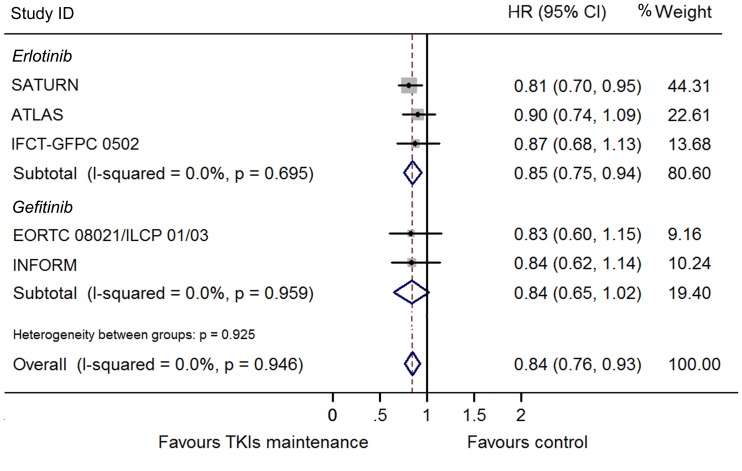
Meta-analysis of hazard ratio (HR) for overall survival (OS).

**Figure 5:**
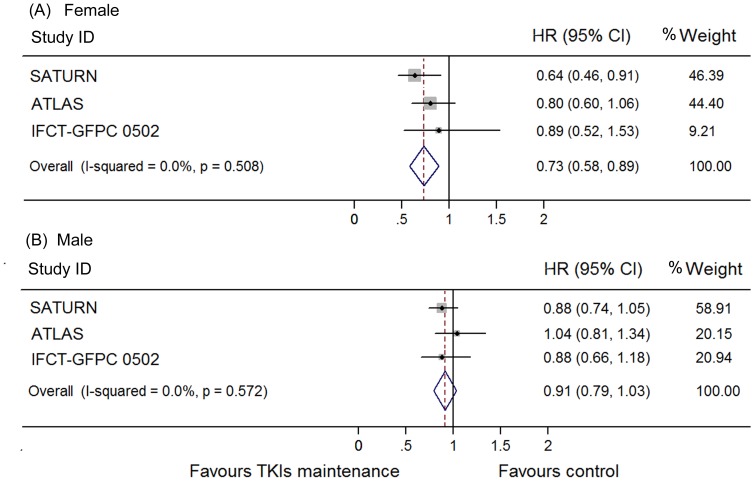
Meta-analysis of hazard ratio (HR) for overall survival (OS) according to sex. (A) Female. (B) Male.

**Figure 6:**
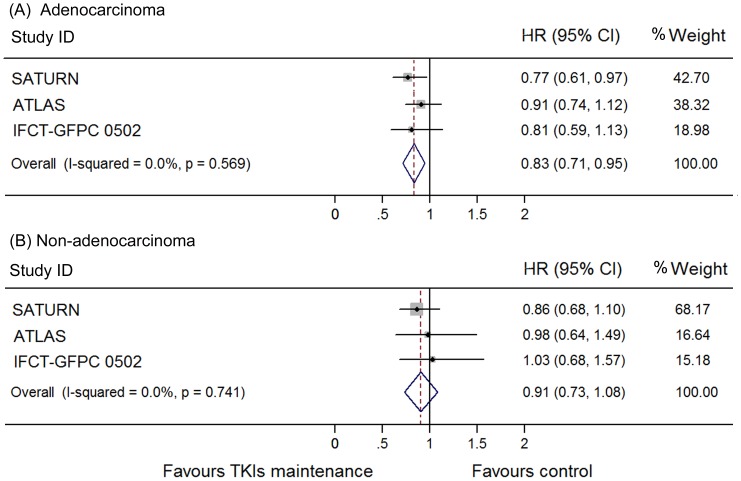
Meta-analysis of hazard ratio (HR) for overall survival (OS) according to histology. (A) adenocarcinoma. (B) non-adenocarcinoma.

**Figure 7:**
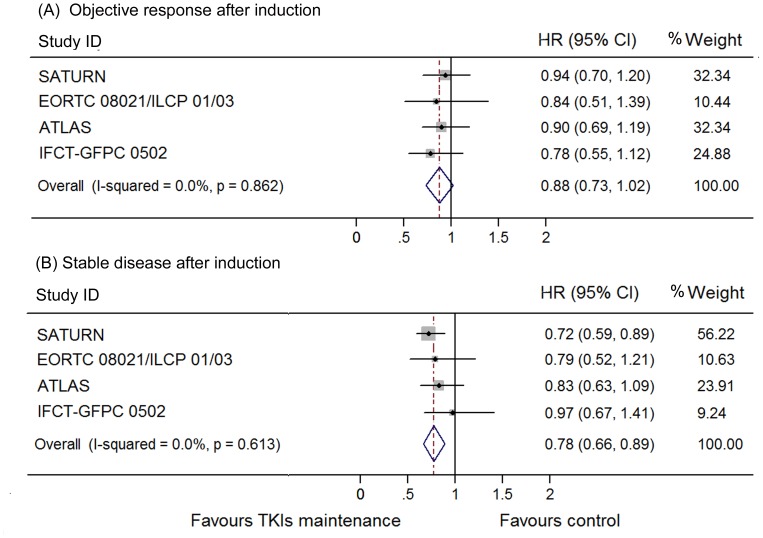
Meta-analysis of hazard ratio (HR) for overall survival (OS) according to previous response to induction chemotherapy. (A) Objective response after induction. (B) Stable disease after induction.

**Figure 8:**
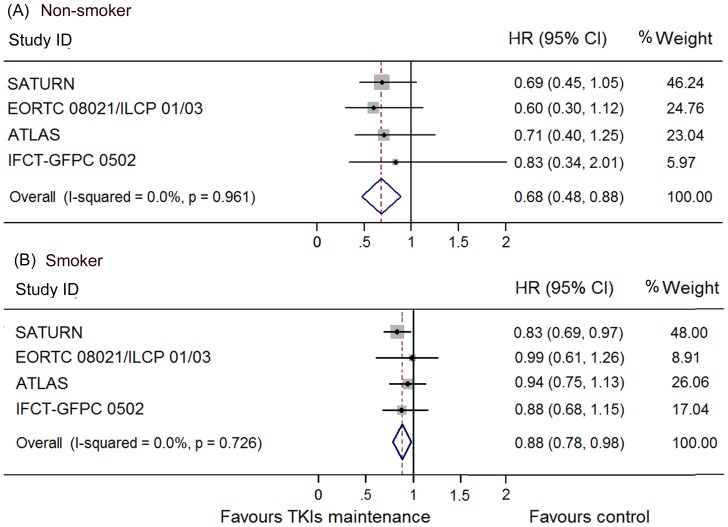
Meta-analysis of hazard ratio (HR) for overall survival (OS) according to smoking status. (A) Non-smoker. (B) Smoker.

The heterogeneity between these trials regarding OS was not significant (I^2^ = 0.0%, p = 0.95) and thus we used a fixed model to pool the HRs. A significant improvement of OS was found in the maintenance group (HR 0.84, 95% CI 0.76–0.93; Fig. 4). This was also shown in the erlotinib subgroup (HR 0.85, 95% CI 0.75–0.94) but not in gefitinib subgroup (HR 0.84, 95% CI 0.65–1.02). To determine whether this result was heavily influenced by SATURN study, the only trial that showed significant OS benefit in TKI maintenance group., a pooled HR without SATURN study was also calculated. A significant improvement of OS was still detected in the maintenance group (HR 0.87, 95% CI 0.76–0.98), but not in the erlotinib (HR 0.84, 95% CI 0.65–1.02) or gefitinib subgroup (HR 0.89, 95% CI 0.75–1.03). Subgroups analysis according to clinical features as female sex, adonocarcinoma, non-smoker, smoker and stable disease to previous induction chemotherapy conferred OS benefit by EGFR TKIs maintenance, while patients who were male, non-adenocarcinoma, and responded to chemotherapy did not (Fig. 5–8). Subgroup analysis regarding ethnicity and EGFR mutation status were available only in SATURN and ATLAS study. We thus did not pool the HRs for these subgroups.

### Data on Harms and Quality of Life

As the adverse events (AEs) were generally less frequent and reported differently, we were unable to carry out a pooled analysis. The most frequent AEs≥grade 3 were rash and diarrhea, which were more frequent in clinical trials on erlotinib. The incidence of ≥grade 3 rash was 9% in the SATURN study, and 10.4% in the ATLAS. The incidence of ≥ grade 3 diarrhea was 2% in the SATURN study and 9.3% in the ATLAS. Assessment of quality of life (QoL, by Functional Assessment of Cancer Therapy-Lung instrument) was obtained from two studies (INFORM and SATURN). In the INFORM study, a significantly higher percentage of patients had a sustained clinically relevant improvement in lung cancer symptoms with gefitinib than with placebo. No significant difference in QoL was found for patients receiving erlotinib compared with those receiving placebo in SATURN study.

## Discussion

The studies on maintenance therapy in advanced NSCLC are heterogenous regarding to study design and compounds, but in general the results are promising for this group of patients. Importantly, the discussion on the trade-off between a gain in PFS or OS versus the toxicity has called for less toxic substances [18], [19]. Recently the TKIs have shown activity in phase III studies of unselected populations with advanced NSCLC with the combined advantage of less toxicity and hospital admittance and the ease of oral administration. Here we performed a meta-analysis on all phase III studies on maintenance TKI treatment where patients were included after the induction chemotherapy, thus the data will more correctly refect the benefit or harm from maintenance therapy. The results of our meta-analysis confirmed that EGFR TKIs (gefitinib or erlotinib) maintenance therapy could provide both PFS and OS benefit in patients with advanced NSCLC who had not progressed after first line chemotherapy. According to the pooled results, EGFR TKIs produced a reduction of 37% and 16% in the risk of progression and death, respectively. Notably, though maintenance arms in all of the studies showed significant benefit in PFS, a significant OS benefit was only found in the SATURN study. Among the five included studies, four were aimed to detect the PFS benefit in maintenance groups and thus the study samples were probably not large enough to detect the difference in OS. Also, the widespread use of second and third line treatment at progression makes the value of OS as end-point more obscure. The EORTC 08021/ILCP 01/03 [8] study, which planed to enroll 598 randomized patients and observe 514 deaths, was designed to test the OS difference, but was prematurely closed due to low accrual after inclusion of 173 patients. This meta-analysis, by pooling the similar randomized control studies, showed strong evidence of OS benefit in EGFR TKI maintenance therapy, both in the erlotinib subgroup and in total.

Clinical and biomarker guided therapy has convincingly been demonstrated in the use of EGFR TKIs as both second line [20], [21], [22] and first line [23] treatment. But this has not been fully defined in maintenance therapy. We thus carried out a subgroup analysis to explore the sub-populations who may benefit or benefit more from maintenance therapy. Interestingly, the PFS benefit existed in all the subgroups including stage, sex and ethnicity, PS, smoking status, histology, EGFR mutation status and previous response to chemotherapy. Patients with stage IIIB or IV, PS 0 or 1, objective response or stable to previous chemotherapy had similar benefit with regard to PFS. Consistent with previous studies in first line, patients who were Asian (HR 0.40, 95% CI 0.21–0.58), female (HR 0.61, 95% CI 0.46–0.75), non-smoker (HR 0.53, 95% CI 0.31–0.75), those who had adenocarcinoma (HR 0.59, 95% CI 0.38–0.79) and most impressively, EGFR mutant (HR 0.12, 95% CI 0.03–0.21) may benefit more from EGFR TKIs. Moreover, a significant OS benefit was also found in patients who were female (HR 0.73, 95% CI 0.58–0.89), nonsmoker (HR 0.68, 95% CI 0.48–0.88), and smoker (HR 0.88, 95% CI 0.78–0.98) or had adenocarcinoma (HR 0.83, 95% CI 0.71–0.95), according to subgroup analysis on OS. In the EORTC study 54/173 patients were recruited in Cairo, Egypt, and potentially this could affect the result if ethnic Egyptians had a higher rate of EGFR mutated tumors. To our knowledge, there are no available data on this. A large study on EGFR mutation status in African-Americans showed no difference in frequency of mutational status, but there is still very little known about other ethnicities than Asian and Caucasian [24].

Although only 27% of patients in INFORM and 59% in SATURN were available for the EGFR mutation analysis, the impressive HR in EGFR mutant positive patients reinforce the notion that EGFR mutation is a predictive biomarker for EGFR TKIs. In the EORTC study, there were not enough cases tested for EGFR to validate these findings. A series of studies [23], [25], [26], [27] have clearly proved the rationale of first line EGFR TKIs in advanced NSCLC patients harboring EGFR active mutation. Since EGFR TKIs can prolong the PFS and OS as maintenance after first line chemotherapy in the same population, then, which strategy will lead to better outcome? As the available data in EGFR TKIs maintenance are still limited, especially the data with EGFR mutation status, future studies should address this question.

Nevertheless, the pooled analysis showed that patients with EGFR wild type also benefit from TKI maintenance (this mainly due to the effect of erlotinib in SATURN). As SATURN is the only trial that showed significant PFS benefit (HR 0.77, 95% CI 0.61–0.97, p = 0.0243) in patients with EGFR wild type, the role of EGFR TKIs in this population should be verified by more studies. An ongoing study conducted by Roche (NCT01328951) may contribute to this issue. This double-blind, placebo-controlled study will evaluate the benefit of first-line maintenance erlotinib versus erlotinib at the time of disease progression in patients with advanced non-small cell lung cancer (NSCLC) who have not progressed following four cycles of platinum based-chemotherapy. Importantly, this study enrolls patients whose tumor does not harbor an EGFR activating mutation.

Another controversial issue is whether we can select suitable patients for maintenance treatment according to the response to first-line treatment. The results are conflicting. In SATURN trial, patients who had stable disease after chemotherapy had a higher OS from maintenance treatment (HR 0.72, 95% CI 0.59–0.89), compared to those who responded to chemotherapy (HR 0.94, 0.74–1.20). Although no similar statistically significant difference in the OS benefit was observed in other studies, this tendency was also found in EORTC 08021/ILCP 01/03 and ATLAS study. The pooled results of the three trials showed that patients who experienced stable disease after first line treatment had a more pronounced OS benefit (HR 0.76, 95% CI 0.64–0.88) than those who gained objective response (HR 0.91, 95% CI 0.74–1.07). However, both categories of patients can achieve similar PFS benefit from EGFR TKIs maintenance (HR 0.66, 95% CI 0.49–0.83 for SD patients and HR 0.65, 95% CI 0.48–0.82 for patients who respond to chemotherapy).

A great advantage of EGFR TKIs is the less toxicity and easy administration, which reinforce the feasibility of EGFR TKIs as maintenance treatment. The summary of the adverse events indicated the incidence of grade 3 or more AEs were generally very low and most patients did not require dose reductions or interruptions.

To strengthen the results of the present meta-analysis, the inclusion criteria were strictly set. Phase II randomized studies were excluded and the randomization had to be done only before the beginning of maintenance so that the effect of the maintenance therapy could be clearly evaluated. The included studies were quite similar in design. Except in ATLAS study, the control groups were placebo (four studies) or observation (IFCT-GFPC 0502). Bevacizumab was previously recommended to use continuously after combination with chemotherapy in advanced NSCLC [28], [29]. In the ATLAS study, bevacizumab was added to the first line chemotherapy, and continued in both maintenance and control groups. The only difference was the maintenance use of erlotinib. Thus, this study was regarded as eligible and was also included in the erlotinib maintenance meta-analysis [10], [11]. The similarity in designs of included studies was important to confer this meta-analysis greater reliability. However, there are some limitations of the present meta-analysis that should be noted. First, the analysis is not based on individual patient data which could have provided further insight into the efficacy of the maintenance strategy. Then, although the included studies are very similar in structure, mechanisms of effect and clinical efficacy, differences on safety profile and clinical effects are also observed between gefitinib and erlotinib. Ideally, only studies published as full manuscripts in peer-reviewed journals should be included, but due to the lack of eligible studies, and after scrutiny of the quality, data from the ATLAS study published in peer-reviewed meeting abstracts was included. Not all studies provided detailed subgroups analysis, and the sample sizes in subgroups were inevitably underpowered. Finally, one should keep in mind that these trials were all partly funded by pharmaceutical industry, with the inherent conflict of interest and possible bias, but these studies were all of high quality and were the only eligible studies to examine the relevant question. The results of this meta-analysis should therefore be carefully interpreted when used in clinical practice.

### Conclusion

In conclusion, we demonstrate that maintenance therapy with EGFR TKIs (gefitinib and erlotinib) produces a significant PFS and OS benefit for patients with advanced NSCLC compared with placebo or observation. The PFS benefit is also significant in all subgroups including stage, sex, ethnicity, PS, smoking status, histology, EGFR mutation status and previous response to chemotherapy. The toxicity associated with EGFR TKIs maintenance was generally low and well tolerated. These data suggest that maintenance with EGFR TKIs after first line chemotherapy is a good treatment strategy in unselected patients with advanced NSCLC, and an excellent option for patients with EGFR mutation.

## Supporting Information

Table S1 PRISMA 2009 Checklist for this Meta-analysis.
(DOC)

Protocol S1 **Protocol for this systematic review.**
(DOCX)
