# Supplementary material for: Gefitinib or Erlotinib as Maintenance Therapy in Patients with Advanced Stage Non-Small Cell Lung Cancer: A Systematic Review
Source: PLoS One. 2013 Mar 21;8(3):e59314. doi: 10.1371/journal.pone.0059314 (PMC3605444; doi:10.1371/journal.pone.0059314)
Supplement: Protocol S1 — Protocol for this systematic review. (DOCX) [file pone.0059314.s002.docx]

**meta-analysis protocol for:**

**Gefitinib or erlotinib as maintenance therapy in patients with advanced stage non-small cell lung cancer: A systematic review.**

**Objectives:**

Maintenance therapy refers to the use of systemic therapy, either by continuing the primary drug or switch to a new one, in patients who get objective response or stable disease after first line chemotherapy. Recently, EGFR TKIs have aroused great attention in maintenance therapy. Behera et al carried out a meta-analysis focusing on the role of single agent maintenance therapy in patients with advanced non-small cell lung cancer. They included twelve studies (5 meeting abstracts, 7 full manuscripts) and showed that single agent maintenance therapy provided superior OS (HR 0.86, 95%CI 0.80-0.92) and PFS (HR 0.62, 95%CI 0.57-0.67). However, only 4 studies (2 meeting abstracts and 2 full manuscripts) about EGFR TKIs were included. Furthermore, because they emphasized the role of switch and continuation, the outcomes of EGFR TKIs maintenance were not analyzed in detail.

The objective of this systematic review is to determine the role of maintenance EGFR TKIs (gefitinib or erlotinib) in patients with advanced NSCLC and to explore which subgroups of patients who will benefit from EGFR TKIs maintenance.

**Methods: Criteria for considering studies for this review**

**Eligibility criteria for trial design:** Peer reviewed articles or abstracts of randomized controlled phase Ⅲ trials reporting the efficacy of gefitinib or erlotinib as maintenance therapy immediately after the first line chemotherapy in stage ⅢB/Ⅳ NSCLC were considered. Randomization should be done just after the induction treatment, before the maintenance period because then the effect of the maintenance treatment would be more clear, excluding the possible interference of different induction regimes. Both blinded and open label trials were eligible. There was no minimum follow-up time.

**Eligibility criteria for the patient population:** Patients should be pathologically or cytologically diagnosed with NSCLC in stage ⅢB/Ⅳ.

**Eligibility criteria for each intervention and comparator:** Maintenance group could be single or combined with other agents except chemotherapeutics, the control group should be placebo or observation or other agents except chemotherapeutics, such as bevacizumab (due to the recommended regimen for bevacizumab is continuously administration). The difference between maintenance group and control group is only the use of TKI.

**Methods: Search methods for identification of studies:**

**Databases:** Pubmed, the EMBASE and the Cochrane library Meeting abstracts: American society of Clinical Oncology (ASCO) (2007-2012) and World Congress of Lung Cancer (WCLC) (2007-2011)

**Key words:** “gefitinib and maintenance”, or “erlotinib and maintenance”, “non-small cell lung cancer (NSCLC)”

Relevant trials were also checked in the clinical trials website (clinicaltrial.gov). Reference lists of original articles and review articles were also examined for additional literature

Dr. Xiaofeng Chen, Dr. Yiqian Liu and Dr. Yingying Qian independently searched and screened the eligible trials. Renhua Guo and Lingjun Zhu reviewed the process to ensure the eligibility of the trials.

**Methods: Data collection and analysis:**

Final list of eligible trials:

| **Studies** | **First**  **author/**  **year** | **Pts n** | **Ethnicity Caucasian/Asian/**  **Other (%)** | **Age**  **M** |
| --- | --- | --- | --- | --- |
| INFORM | Zhang L 2012 | 296 | 0/100/0 | 55 |
| EORTC 08021/ILCP 01/03 | Gaafar RM 2011 | 173 | NR | 61 |
| SATURN | Cappuzzo F 2010 | 889 | 84/15/1 | 60 |
| IFCT-GFPC 0502 | Perol.M 2012 | 310 | NR | 58 |
| ATLAS | Kabbinavar FF 2010 | 768 | 78/12/10 | 64 |

**Data collection:** Dr. Xiaofeng Chen and Dr. Yiqian Liu will collect the data using the standardized data compilation forms. Disagreements were resolved by discussion. When the data we need are not available from the published paper, Xiaofeng Chen is responsible for contacting the primary investigators to get their help.

**Extracted data:** the first author, year of publication, number of patients, median age, percentage of smoker, percentage of adenocarcinoma, primary endpoint, percentage of known EGFR status, percentage of EGFR mutation and data related to the clinical outcomes such as objective response rate (ORR), progression free survival (PFS), overall survival (OS), adverse events (AE). For PFS, OS, the p value and hazard ration as well as the 95% confidence intervals will be also extracted.

**Predefined subgroups for OS and PFS:** Stage, sex, ethnic, performance status, pathological type, EGFR status, previous response to induction chemotherapy.

**Analysis:** the analysis will not be based on individual patient level. The PFS and OS will be estimated by collection of HRs with 95% Confidence Intervals (CIs) which were mentioned in the original publications. The pooled risk ratio (RR) for ORR will be calculated from the number of events and the number of patients at risk in each group. The heterogeneity of the studies is tested and a P<0.1 defined as heterogenous. A fixed-effect model (Mantel Haenszel) will be applied in case of absence of heterogeneity between studies and otherwise a random-effect model will be performed. The software used is Stata (version 11, Stata, USA).
